# Behavioral report of *Chrysomya rufifacies* in response to substrate infestation by *Lucilia sericata* and *Lucilia cuprina* using a tetrahedron olfactometer device

**DOI:** 10.3389/finsc.2024.1385884

**Published:** 2024-06-14

**Authors:** Alicia Fonseca-Muñoz, Gregorio Hernández-Salinas, Salvador Ordaz-Silva, Imelda Virginia López-Sánchez, Jorge Luis Delgadillo-Ángeles, Evert Villanueva-Sánchez, Clemente Villanueva-Verduzco, Nadia S. Gómez-Domínguez, Carlos Granados-Echegoyen

**Affiliations:** ^1^ Universidad Autónoma Benito Juárez de Oaxaca, Facultad de Sistemas Biológicos e Innovación Tecnológica, Oaxaca, Mexico; ^2^ Tecnológico Nacional de México/Instituto Tecnológico Superior de Zongolica, Veracruz, Mexico; ^3^ Universidad Autónoma de Baja California, Facultad de Ingeniería y Negocios San Quintín, Baja California, Mexico; ^4^ Consejo Nacional de Humanidades, Ciencia y Tecnologia (CONAHCYT)-Universidad Autónoma Chapingo, Laboratorio Nacional de Investigación y Servicio Agroalimentario y Forestal, Estado de México, Mexico; ^5^ Departamento de Fitotecnia, Universidad Autónoma Chapingo, Estado de Mexico, Mexico; ^6^ Instituto Politécnico Nacional, (CEPROBI) Centro Desarrollo de Productos Bióticos, San Isidro, Morelos, Mexico; ^7^ CONAHCYT- Instituto Politécnico Nacional, Centro Interdisciplinario de Investigación para el Desarrollo Integral Regional (CIIDIR) Unidad Oaxaca, Santa Cruz Xoxocotlán, Oaxaca, México

**Keywords:** non-attraction, *Chrysomya*, food resource, ecology behavior, Calliphoridae

## Abstract

Calliphoridae are widespread globally and can inhabit a variety of habitats. In this brief report, we assessed the appeal of *Chrysomya rufifacies* to resources that were previously inhabited by *Lucilia sericata* and *L. cuprina*, both common carrion colonizers. Two hundred adult male and female (1:1) *C. rufifacies* were released under controlled conditions into clear plastic cages (45 x 45 x 45 cm) with four types of substrates: beef-liver; liver with 100 third-instar larvae of *L. cuprina*; (III) liver containing 100 third-instar larvae of L. sericata; and liver containing 100 third-instar larvae of *C. rufifacies*. Each substrate was left in place for 24 hours at the end of a tube connected to the cage, where sticky traps were positioned to capture flies that might have been attracted to a specific substrate. The results indicate variations in the attraction of flies to different types of livers colonized by larvae of various species. It is suggested that flies may have specific preferences depending on the species of larvae present in the substrate. The liver without larvae was the preferred choice, while beef liver with *C. rufifacies* larvae was the least attractive. Results of statistical tests indicated that there is independence between attractiveness preference and the presence of *C. rufifacies* flies. Although there is a trend among certain levels of the variables in the correspondence analysis, these relationships are not statistically significant. However, they indicate specific patterns of association between different groups of flies and species of larvae. This study demonstrated that *C. rufifacies* does not show reduced attraction to any of the resources. A tetrahedron olfactometer device has been used for the first time in a behavioral study of *C. rufifacies* flies. This may enable future studies to enhance the understanding of fly behavior.

## 1 Introduction

Several arthropods are attracted to carrion within minutes of death (1, 2). The attraction of these arthropods is influenced by various cues, including odor (1, 3). Many of the insects that colonize vertebrate carcasses are important for forensic investigations. Acceptance, i.e., oviposition, by these insects is an important part of the decomposition process (4). Colonization occurs in the natural cavities of the body, such as the nose, mouth, and ears. These are influenced by the nutritional value of resources (5), including feces (6), attractants such as chicken and beef livers (7), human blood, semen, and saliva (8), and others. Sometimes, predators are guided by cues indicating the presence of prey, which can alter their abundance and behavior (9). Some signals used by members of the same species and predators are excretions and secretions produced by blowfly larvae (10). Some adult blow flies (Diptera: Calliphoridae) do not rely on carrion for sustenance. Their competition is influenced by both density-dependent and density-independent factors, which occur during the larval stage (11).

The Calliphoridae family is distributed worldwide and is studied for its significance as forensic evidence associated with decomposing remains (1). The genera *Calliphora, Cochliomyia, Chrysomya*, and *Lucilia* are the most prevalent blowflies within the Calliphoridae family. In some regions of the world, *Chrysomya rufifacies* have been identified as the primary blowfly species that colonizes carrion (2, 12). The hairy maggot blowfly is found in tropical and subtropical regions worldwide, including Central and South America (13). The larvae of this species are predatory and cannibalistic during the second and third instars. These behaviors may provide competitive advantages over both larvae from different species and larvae from the same species (13). Such selection has influenced the behavior of other *Chrysomya* species competing for the same resources. Yang and Shiao (14) reported that female *C. megacephala* avoided laying eggs in the presence of *C. rufifacies* larvae; however, *C. rufifacies* showed a preference when *C. megacephala* larvae were present. Giao and Godoy (15) suggested that predation by other species may influence oviposition behavior in other calliphorids.

Other important calliphorids in forensic investigations include *Lucilia sericata* and *L. cuprina*. They are reported to be the first colonizers of corpses and are forensically important (1, 16). Sherman (17) and other studies have reported that they also cause myiasis. However, *L. sericata* larvae are also used for debriding necrotic ulcers in maggot therapy (18). Oviposition behavior varies among blowfly species. Limited research has been conducted on the relationship between other species and predators in their larval stage. There has been a growing interest in understanding the attraction of flies to a food source colonized by third-stage blowfly larvae. Forensic entomologists need to understand the behavioral ecology of adult blowflies (10). However, there is limited knowledge about attracting adults of *C. rufifacies* to third-stage larvae of *L. sericata, C. rufifacies*, and *L. cuprina*. This brief report aims to investigate the behavior of adult *C. rufifacies* towards a food source colonized by the larvae of these blowflies.

## 2 Materials and methods

### 2.1 Blow flies

Blowflies were collected from a decomposing pig head in Santa María, Tule, Oaxaca, Mexico (17° 02’50” N, 96° 38’00” W). Maggots were collected and placed in 1000 mL plastic containers filled with sterile sawdust, which were then covered with white plastic sheets. The resulting adult flies were identified using the method described by Whitworth (19). They were then transferred to 30 x 30 x 30 cm plastic cages and kept under controlled conditions (26 ± 1°C, 70 ± 10% relative humidity, and a 12:12 photoperiod). Blowflies were fed 50 g of bovine liver, sugar, and water ad libitum for the first five days after emergence. Oviposition of the flies was induced by placing 50 grams of beef liver in gravid blowfly cages. The eggs were placed in 1-liter plastic jars filled with sawdust (20). After hatching, they were fed 300 grams of bovine liver. All flies were used from generations F2 to F4. The pupae were removed and transferred to 30 cm³ plastic cages to complete their development into adulthood.

### 2.2 Attraction bioassays

**Figure 1:**
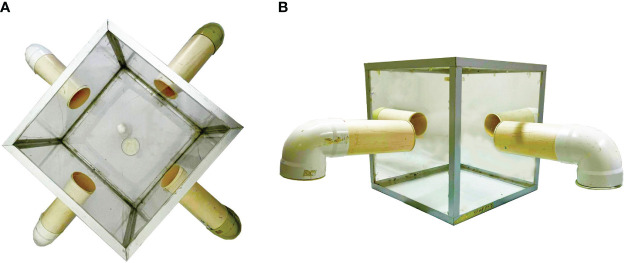
A tetrahedron olfactometer device for quantification of study variables of *C. rufifacies* to various food substrates colonized by larvae of *Lucilia sericata* and *Lucilia cuprina*. **(A)** Zenithal perspective, **(B)** Frontal view.

Adult *C. rufifacies* flies were maintained in 30 cm³ plastic cages and provided with granulated sugar and water *ad libitum*. The flies were fed cow liver blood for the first 5 days after emerging. A total of 200 adult *C. rufifacies* flies, 7 days old, were aspirated from their cages using an aspirator (AC/DC Aspirator, BioQuip USA). The method used was modified from that described by Ma et al. (21)Tomberlin et al. (22); and Flint and Tomberlin (23). In the present study, we assembled and used a tetrahedron olfactometer device. We transferred these flies to a modified 45 x 45 x 45 cm clear plastic cage. The cage had four holes in the center of each wall. We placed a 4-inch diameter PVC tube with a perforated lid inside the cage, with each hole measuring 0.4 mm in diameter (**Figure 1**). Four types of substrates were studied: (I) liver; (II) liver containing 100 third instar larvae of *L. cuprina*; (III) liver containing 100 third instar larvae of *L. sericata*; (IV) liver containing 100 third instar larvae of *C. rufifacies*. To avoid positional bias, we allowed the cages to dry for 24-48 hours between experiments after thorough cleaning. Additionally, we rotated the tubes to different positions to ensure varied substrate exposure. We placed each substrate at the end of a tube connected to the cage and left it in position for 24 hours. Sticky traps (Trapper Max-Free, Bell Laboratories, Madison, WI, USA) were placed to facilitate the capture of flies exhibiting substrate preference. We recorded the number, sex, and gravid status of the captured flies.

### 2.3 Statistical analysis

A randomized experimental design was utilized. Five cages were set up as replications and the number of flies was counted twenty times in each cage for males, non-gravid females, and gravid females. The Kolmogorov-Smirnov test was used to assess the distribution of the data, while the chi-square test was used to assess the relationship between the variable’s “groups” and “species”. A simple correspondence analysis was conducted to assess the relationship between two variables or their levels and a multinomial logistic regression analysis was employed to model the behavior of the species to the variable’s “groups” and “species”. In all tests, a significance level of α = 0.05 was utilized with the JMP statistical software v7.0.

## 3 Results

**Figure 2:**
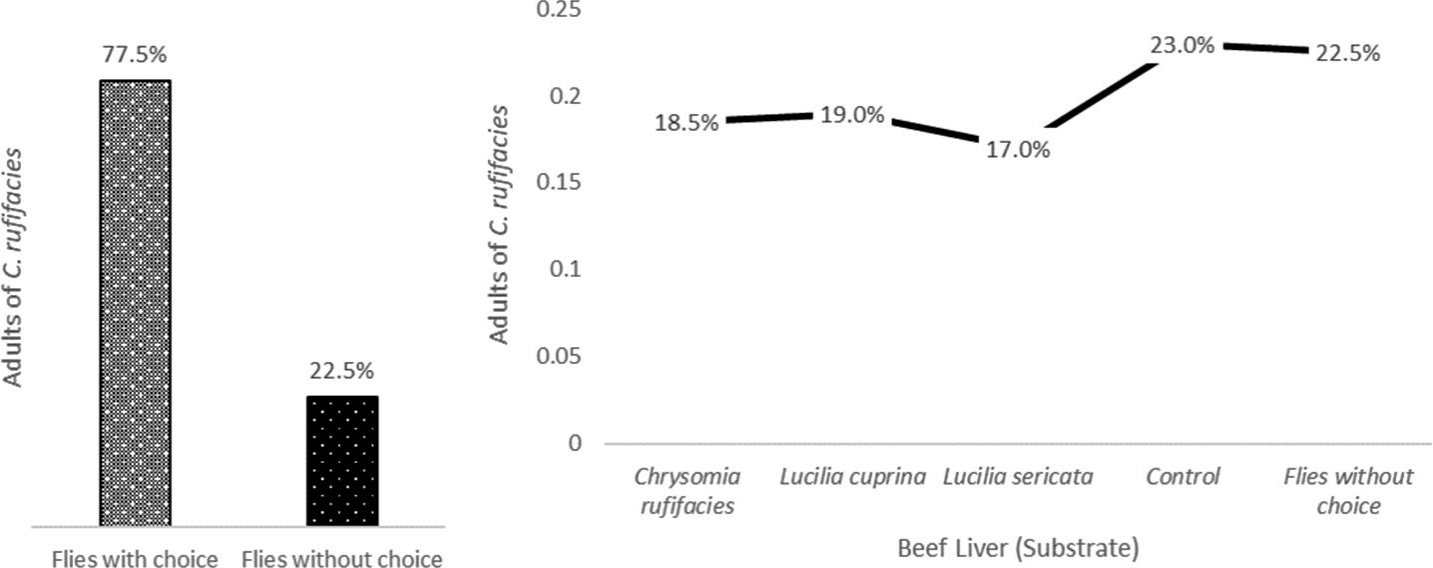
Attraction of *Chrysomya rufifacies* to Different Substrates Colonized by *Lucilia sericata* and *Lucilia cuprina* Larvae.

Variation in the attraction of flies to different types of livers colonized by larvae of different species is evident. This suggests that flies may have specific preferences depending on the species of larvae found in the substrate. Several factors could influence fly feeding preferences, including odor, texture, chemical composition, and the presence of associated microorganisms. We observed that Substrate I, the control treatment, appears to be the preferred choice of the flies, as it has the highest frequency and the highest attraction percentage. This could be because flies prefer the liver without larvae as a food source. In the case of beef liver with *C. rufifacies* larvae (Substrate IV), this treatment exhibited the lowest frequency and attraction percentage. It seems that flies are less attracted to beef liver colonized with *C. rufifacies* larvae compared to the other options. Similarly, beef liver with *L. cuprina* larvae (Substrate II) showed a slightly higher frequency and attraction than the *C. rufifacies* larvae treatment, but still lower than the control. The flies seem to exhibit a moderate preference for this type of liver colonized by *L. cuprina* larvae. For beef liver with *L. sericata* larvae (substrate III), similar to the treatment with *C. rufifacies* larvae, this treatment exhibits lower frequency and attractiveness compared to the control and the liver colonized by *L. cuprina* larvae. The data presented in the results provide an intriguing insight into the feeding preferences of flies regarding various types of beef livers colonized by larvae of three different species (**Figure 2**).

**Figure 3:**
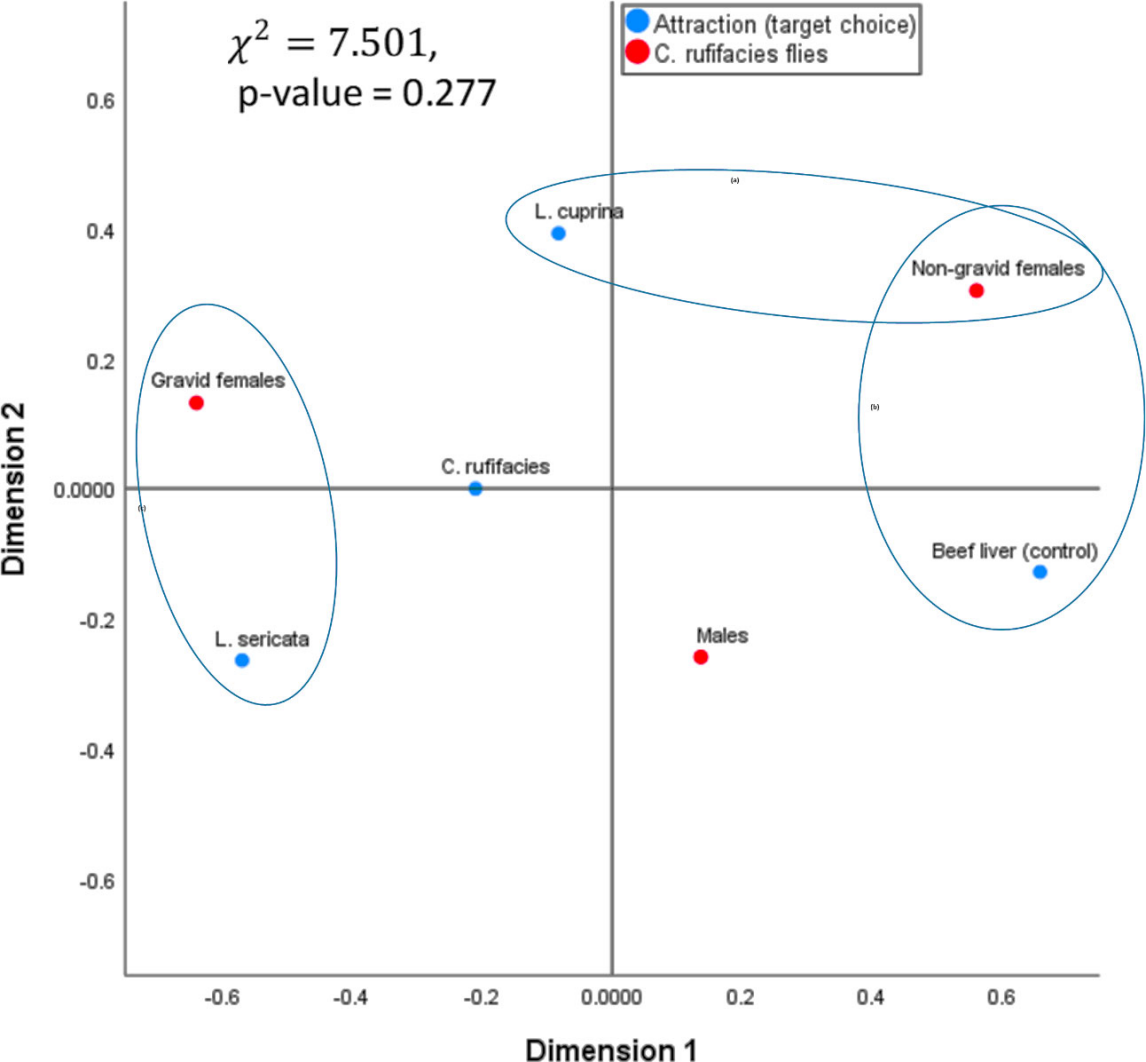
Correspondence analysis of *C. rufifacies* to various food substrates colonized by larvae of *Lucilia sericata* and *Lucilia cuprina*.

The results of the statistical test, based on the Chi-square test, indicate a p-value greater than 0.05. Therefore, variables are independent or have no dependence between variables. That is, one does not affect the other, and vice versa. The results of the statistical test indicate that the two variables, attraction (target choice) and *C. rufifacies* flies, are independent of each other (**Table 1**). In correspondence analysis, a specific trend is observed in the graph between certain levels of each variable to the other variable, which is indicated by ellipses. Although these relationships or dependencies are not statistically significant, it is observed that some groups of both variables are relatively close to each other, while others are further away from the center. For instance, the group of “gravid females” from one of the two variables is similar to the *“L. sericata”* group from the other variable. Therefore, it could be argued that while the statistical test results show no significant relationship or dependency between the two variables, a discernible tendency or pattern indicates that gravid females are commonly associated with the species *L. sericata*. In a similar vein, the “non-gravid females” exhibit a similar pattern, with two distinct groups or subgroups identified: one associated with the “beef liver (control)” and the other with *L. cuprina*. Therefore, the farther it is from the center, the greater its weight and potential influence on, or dependence on, other levels or groups of variables (**Figure 3**).

**Table 1:** Abundance (N) and relative abundance (%) of adult *Chrysomya rufifacies*, categorized by sex, attracted to resources colonized by *Lucilia sericata* and *Lucilia cuprina* larvae.

Treatment (Beef Liver with blowflies)	Groups of adult *C. rufifacies* ^a^			Chi-square test^b^	
	Males	Non-gravid females	Gravid females	χ2	p-value
Control^c^ (I)	23 (11.5%)	15 (7.5%)	8 (4.0%)	7.499	0.484
L. cuprina (II)	15 (7.5%)	10 (5.0%)	13 (6.5%)		
L. sericata (III)	15 (7.5%)	5 (2.5%)	14 (7.0%)		
C. rufifacies (IV)	16 (8.0%)	8 (4.0%)	13 (6.5%)		
Unrecovered flies	20 (10.0%)	11 (5.5%)	14 (7.0%)		

^a^Number of adult *C. rufifacies* flies detected in larval treatments and control. ^b^Significance level of α=0.05 in hypothesis testing. ^c^Beef liver without larvae, as control.

## 4 Discussion

This study demonstrates that blowflies do not exhibit differences in attraction to food colonized by both species. However, this response was only observed in third-instar maggots and not in first or second-instars. Distance may also be a factor. Previous behavioral studies with *C. rufifacies* have shown that it preys on *C. macellaria*, possibly due to its weaker competitive abilities compared to *C. rufifacies* (24). However, there are other behavioral studies of adult *C. rufifacies*, especially concerning the presence of Calliphoridae species.

Wall and Fisher (25) discussed the utilization of their eyes by *L. sericata* for locating and seeking scents to lay eggs over short distances. However, some calliphorids responded to distances greater than 10 meters (26). In our study, we observed attraction at a short distance of about 45 centimeters, where adult *C. rufifacies* flies were attracted by the presence of third-instar larvae of blowfly species. There was no significant difference between females and males in terms of attractiveness to *C. rufifacies* for colonizing food resources. Gomes et al. (27) suggested that olfactory cues may stimulate blowflies to oviposit. However, visual cues are the key factors that influence flies’ decisions on where to oviposit.

Competition for resources can lead insects to develop various survival strategies, such as predation and cannibalism. The signals that indicate to a scavenger insect that the presence of a resource is beneficial to its offspring are not well understood in scavenging insects. We endorse the study conducted by Tomberlin et al. (28), which highlights the significance of *C. rufifacies* in forensic entomology due to its larval biology. The study reveals that during the initial larval stage, *C. rufifacies* feeds directly on carrion, while in the second and third stages, they act as facultative predators of larvae of other species. Therefore, our study aims to investigate the relationship between the presence of flies and the larvae of other species such as *L. sericata* and *L. cuprina*.

Yang and Shiao (14) reported that female *C. megacephala* avoids laying eggs in the presence of *C. rufifacies* larvae. However, *C. rufifacies* showed a preference for oviposition in the presence of *C. megacephala* larvae. The study did not demonstrate a preference for oviposition by *C. rufifacies* when in the presence of *L. sericata* and *L. cuprina* species compared to the control group. On the other hand, Shah and Sakhawat (29) demonstrated that calliphorids exhibit preferences for different colors and stages of meat. In our study, we utilized beef liver at 24 hours of decomposition, which elicited attraction at 26 ± 1°C, 70 ± 10% relative humidity, and a 12:12 photoperiod. Previous studies have shown that *C. macellaria* selectively oviposits in beef liver resources with *C. rufifacies* larvae more than with *C. macellaria* (10). However, in the present study, the oviposition rate of *C. rufifacies* was similar between *L. cuprina* and *L. sericata.*

Although the correlation coefficient indicates a low correlation between the groups and the species present, the simple correspondence analysis revealed a tendency for “gravid” flies to be associated with *L. sericata* larvae, while “males” were more commonly found in the “control” group. However, there was no significant tendency, dependence, or statistical association with the females of *C. rufifacies* or with the presence of *L. cuprina* larvae and *C. rufifacies*. In the study, 43% of males showed a stronger reaction in the liver without larvae of any species. This indicates that the most represented gravid females were attracted by larvae of *L. sericata*, with a 30.7% response rate. The presence of non-gravid females in all three treatments was consistent, ranging from 42% to 44%. However, the control group was smaller. The study revealed that adult *C. rufifacies* were more attracted to food that had been colonized by larvae of *L. sericata*, unlike their species. Studies conducted with other species (predator or prey) suggest that blowflies may exhibit escape or attraction behavior towards a previously colonized resource. This study demonstrated that *C. rufifacies* did not exhibit reduced attractiveness to such resources.

## 5 Conclusions and future directions

This brief communication is part of a project led by the primary author in collaboration with a multidisciplinary team of researchers in Mexico. The project aims to understand the biology and ecology of flies that are of forensic significance in the country. The purpose of this report is to conduct a more in-depth investigation into the chemical and physical characteristics of each type of substrate and their impact on the attraction of calliphorid flies. Our findings may offer insight into how these variables influence the preference of flies for a specific substrate.

Understanding the feeding preferences of flies is crucial for managing pests in agricultural, livestock, and urban settings. The results obtained could have practical implications for the development of more effective pest control strategies, such as the selection of baits or traps tailored to specific fly species. In addition, they provide a solid foundation for future research on the ecology and feeding behavior of flies, as well as the development of more effective pest control strategies, which will be detailed in future publications. This study demonstrated that *C. rufifacies* does not show reduced attraction to any of the resources.

## Data availability statement

The original contributions presented in the study are included in the article/supplementary material. Further inquiries can be directed to the corresponding author.

## Author contributions

AF-M: Writing – review & editing, Writing – original draft, Visualization, Validation, Software, Resources, Project administration, Investigation, Conceptualization. GH-S: Writing – review & editing, Visualization, Validation, Supervision, Software. SO-S: Writing – review & editing, Visualization, Validation, Supervision, Software, Resources. IL-S: Writing – review & editing, Visualization, Validation, Supervision, Software. JD-Á: Writing – review & editing, Visualization, Validation, Supervision, Software. EV-S: Writing – review & editing, Visualization, Validation, Supervision, Software, Investigation. CV-V: Writing – review & editing, Visualization, Validation, Supervision, Software. NG-D: Formal analysis, Methodology, Software, Validation, Visualization, Writing – review & editing. CG-E: Writing – review & editing, Writing – original draft, Visualization, Resources, Project administration, Methodology, Investigation.
